# Cystic and alveolar echinococcosis: Successes and continuing challenges

**DOI:** 10.1371/journal.pntd.0005477

**Published:** 2017-04-20

**Authors:** Christine M. Budke, Adriano Casulli, Peter Kern, Dominique A. Vuitton

**Affiliations:** 1 Department of Veterinary Integrative Biosciences, College of Veterinary Medicine & Biomedical Sciences, Texas A&M University, College Station, Texas, United States of America; 2 WHO Collaborating Centre for the epidemiology, detection and control of cystic and alveolar echinococcosis (in humans and animals); European Reference Laboratory for Parasites; Istituto Superiore di Sanita’, Rome, Italy; 3 Department of Internal Medicine III, University Hospital of Ulm, Ulm, Germany; 4 WHO-Collaborating Centre on Prevention and Treatment of Human Echinococcosis, and French National Reference Centre for Echinococcoses; University Bourgogne Franche-Comté and University Hospital; Besançon, France; Baylor College of Medicine, Texas Children's Hospital, UNITED STATES

Cystic and alveolar echinococcosis are diseases of animals and humans caused by the larval stage of tapeworms in the genus *Echinococcus*. Cystic echinococcosis (CE), caused by *E*. *granulosus* sensu lato and alveolar echinococcosis (AE), caused by *E*. *multilocularis*, have a substantial public health impact globally. Both conditions are considered neglected tropical diseases (NTDs) and neglected zoonotic diseases (NZDs), and prioritized by the World Health Organization (WHO) (http://www.who.int/neglected_diseases/diseases/en/). Within the last 10 years, advancements have taken place in *Echinococcus* biology and genetics, including the delineation of new species within *E*. *granulosus* sensu lato [1] and full sequencing of *E*. *granulosus* sensu stricto and *E*. *multilocularis* [2]. Consequently, researchers are now provided with new tools to better understand parasite biology and host-parasite interactions, with the goal of opening new avenues for therapy [2].

Although often discussed together, CE and AE are two distinct chronic diseases, with CE considered mainly a disabler, while AE is fatal if left untreated [3]. Clinical manifestations of human CE typically result from the growth of single or multiple cysts in the liver, lungs, and/or other organs that can eventually produce a mass effect and impair organ function. Treatment usually consists of a watch-and-wait approach, albendazole administration, and/or surgical (general or percutaneous/perendoscopic) intervention, depending on the stage, integrity, number, size, and location of cysts [4]. In contrast, AE acts more like an invasive tumor, which manifests predominantly in the liver, but can infiltrate adjacent organs and tissues and produce distant metastases [3, 5]. Cure can be achieved by radical surgery, including ex-vivo liver resection [6]. However, most patients are not surgical candidates at the time of diagnosis and receive life-long albendazole therapy [3, 7, 8].

A substantial obstacle to controlling CE and AE is lack of data on the number of cases locally and globally. During the last decade, great strides have been made in improving the amount and quality of collected case data. Large-scale census studies have been conducted in China since the 1990s [9]. The first formal initiative to collect data on human AE was the European Echinococcosis Registry (EurEchinoReg), launched in 1997 [5]. Recently, the European Commission funded a collaborative and translational project (HERACLES: Human cystic Echinococcosis ReseArch in CentraL and Eastern Societies; http://www.heracles-fp7.eu/) to assess the ultrasound prevalence of CE in rural areas of Eastern Europe and adjacent countries, create an online prospective multi-center European Register of CE cases (ERCE) [10], and improve available serological and therapeutic tools as well as molecular diagnostics.

Building on enhanced case registration, strides have been made towards obtaining improved estimates of the burden of both CE and AE. Initial global estimates are now available for both conditions [11, 12]. More recently, two large-scale burden of disease initiatives, the global burden of foodborne diseases project overseen by the WHO and the Global Burden of Disease (GBD) Study, have included Disability Adjusted Life Year (DALY) estimates for echinococcosis. The WHO study estimated an echinococcosis (AE and CE combined) burden of approximately 871,000 DALYs in 2010. Of this total, approximately 184,000 DALYs (equating to 188,000 new cases per year) and 688,000 DALYs (equating to 18,400 new cases per year) were attributable to CE and AE, respectively. For the year 2013, the GBD Study estimated a similar number of DALYs for CE and there is currently no GBD estimate for AE [3]. While there were differences in how these two initiatives approached filling epidemiologic data gaps (e.g., lack of data on infection frequency), the generated preliminary values allow for a starting point from which to improve burden estimates as these gaps are filled.

In order to improve case recording worldwide and ensure the appropriate management of patients, accurate diagnostics are needed. In this regard, the main advances have been achieved by the combination of serological tests and functional imaging to monitor response to treatment [3, 4, 7] and the use of molecular identification and species characterization in difficult cases [13]. Although antibodies against antigen B or antigen 5 show a higher sensitivity, crude parasite extracts (e.g., hydatid fluid) continue to be most widely used for antibody detection for CE cases [13]. Lack of test accuracy remains a critical issue for the diagnosis of CE, especially during community screenings. Despite the use of the highly *E*. *multilocularis*-specific Em2 and Em18 antigens, cross-reactivity remains a problem and source of misinterpretation in areas where CE and AE co-exist [13].

Over the last decade, a structured cyst stage-specific approach to patient management has been recommended based on the WHO-International Working Group on Echinococcosis cyst classification scheme, which was officially adopted in 2010 [4]. Globally, there has been a push for physicians to take a patient’s clinical condition, technical capability of the healthcare facility, safety and effectiveness of the approach, and the cost of treatment into account when devising a treatment plan, with management of AE especially complex [3, 8]. Unfortunately, for both diseases, prospective studies to guide evidence-based therapeutic strategies are lacking and the adoption of a stage-specific approach by physicians has been slow [3]. In addition, whatever treatment plan is selected, long-term follow-up should be mandatory in order to detect recurrences [3, 4].

Control program implementation for CE has historically relied on a combination of local education regarding slaughter and hygiene practices and dog population management and/or deworming initiatives using praziquantel. The development of vaccines targeting sheep intermediate hosts has now added a new tool to combat CE in highly impacted populations. The EG 95 vaccine has been tested in a number of countries, including China and Argentina with promising results [9, 14]. Control program evaluation relies on surveillance for the pathogen in domestic dogs and ruminants, which is costly and labor-intensive. Overall, the prevalence of human CE has been reduced in areas where long-term intensive control programs have been put in place. However, in most resource-poor areas, CE control remains a challenge [9]. Compared to CE, control measures for AE are more logistically challenging due to the presence of wildlife hosts. Researchers are working to better understand the ecology of wildlife definitive (e.g., foxes and other wild canids) and intermediate (e.g., small mammals) hosts, to predict risks for human infection and improve anthelminthic baiting practices in rural and urban areas [9].

While progress has been made in reporting, diagnosis, treatment, and control, CE and AE are still very much neglected diseases. Albendazole availability and/or cost continue to be a problem in both socioeconomically disadvantaged and high-income countries. In addition, at present, there are no alternative drugs for patients with AE who experienced severe side effects and cannot be treated with albendazole (or mebendazole). Therefore, additional research into new therapeutic agents is needed [2]. The chronic nature of CE and AE and a strong reliance on advanced imaging for diagnosis continues to impact the speed of case detection in socioeconomically disadvantaged communities. Occurrence of AE as an opportunistic infection in patients with therapeutic immune suppression may have worrisome consequences in the near future [3, 15]. Going forward, control will continue to rely on taking a One Health approach, involving physicians, veterinarians, ecologists, social scientists, laboratory scientists, and others. We encourage the scientific community to perform prospective collaborative studies and policymakers to promote new drug development and improved case notification. The echinococcosis community moves into the next decade proud of their achievements, but realizing that there is still much work remaining.
